# Film-Forming Spray of Water-Soluble Chitosan Containing Liposome-Coated Human Epidermal Growth Factor for Wound Healing

**DOI:** 10.3390/molecules26175326

**Published:** 2021-09-02

**Authors:** Abd. Kakhar Umar, Sriwidodo Sriwidodo, Iman Permana Maksum, Nasrul Wathoni

**Affiliations:** 1 Department of Pharmaceutics and Pharmaceutical Technology, Faculty of Pharmacy, Universitas Padjadjaran, Sumedang 45363, Indonesia; nasrul@unpad.ac.id; 2 Biochemistry Laboratory, Department of Chemistry, Faculty of Mathematics and Natural Science, Universitas Padjadjaran, Sumedang 45363, Indonesia; ip_maksum@unpad.ac.id

**Keywords:** human epidermal growth factor, soy lecithin liposome, water-soluble chitosan, film-forming spray

## Abstract

Human epidermal growth factor (hEGF) has been known to have excellent wound-healing activity. However, direct application to the wound area can lead to low hEGF bioavailability due to protease enzymes or endocytosis. The use of liposomes as coatings and carriers can protect hEGF from degradation by enzymes, chemical reactions, and immune reactions. Sustained release using a matrix polymer can also keep the levels of hEGF in line with the treatment. Therefore, this study aimed to develop a film-forming spray of water-soluble chitosan (FFSWSC) containing hEGF-liposomes as a potential wound dressing. The hEGF-liposomes were prepared using the hydration film method, and the preparation of the FFSWSC was achieved by the ionic gelation method. The hydration film method produced hEGF-liposomes that were round and spread with a Z-average of 219.3 nm and encapsulation efficiency of 99.87%, whereas the film-forming solution, which provided good sprayability, had a formula containing 2% WSC and 3% propylene glycol with a viscosity, spray angle, droplet size, spray weight, and occlusion factor of 21.94 ± 0.05 mPa.s, 73.03 ± 1.28°, 54.25 ± 13.33 µm, 0.14 ± 0.00 g, and 14.57 ± 3.41%, respectively. The pH, viscosity, and particle size of the FFSWSC containing hEGF-liposomes were stable during storage for a month in a climatic chamber (40 ± 2 °C, RH 75 ± 5%). A wound healing activity test on mice revealed that hEGF-liposomes in FFSWSC accelerated wound closure significantly, with a complete wound closure on day 6. Based on the findings, we concluded that FFSWSC containing hEGF-liposomes has the potential to be used as a wound dressing.

## 1. Introduction

Various types of growth factor hormones (GFs) have been known to have wound-healing activities [1,2]. In normal circumstances, when a wound occurs, the body responds in several stages, namely: hemostasis, inflammation, proliferation, and remodeling. At the proliferation stage, GFs are released to accelerate wound healing, including vascular endothelial growth factor (VEGF), fibroblast growth factor (FGF), and human epidermal growth factor (hEGF). Among these GFs, hEGF has a function that includes the role of all GFs, including accelerating the regeneration of keratinocyte cells, fibroblasts, and the vascular epithelium [3,4,5]. It has been reported that the healing of diabetic foot ulcers (DFU) with hEGF therapy was five weeks faster than conventional treatment [6]. Research conducted by Tsang et al. (2003) in 21 patients with 0.04% hEGF showed total wound closure after 12 weeks [7]. However, similar to other proteins and polypeptides, the stability of hEGF is poor. Several studies have shown that chronic wounds locally secrete the protease enzyme that breaks down hEGF, which can reduce the effectiveness of its treatment. The protection of hEGF from these proteolytic enzymes has been investigated using antiproteolytic agents. It has been shown to increase the efficacy of hEGF in accelerating wound healing. However, this agent also significantly influences systemic enzymatic metabolism through topical administration [8].

In addition to antiproteolytic agents, the coating method using liposomes can also improve the in vivo stability of drugs. Lipid bilayers in liposomes prevent enzymatic degradation, immune reactions, and chemical interactions [9]. It has been reported that liposome-coated hEGF was more effective at healing wounds than hEGF itself [3]. Increased stability and localized delivery of hEGF in the injured area has also been reported to be better with liposome coating [10,11,12,13,14,15]. Due to its low stability, hEGF with immediate release will also only last for a short time. It has been reported that 60% of hEGF disappears within two hours after administration, which is most likely caused by earlier enzymatic degradation or the mechanism of receptor-mediated endocytosis [16]. One way to deal with these problems is through sustained release using a matrix, such as a film-forming spray preparation, which can increase the bioavailability of drugs with a low incidence of irritation compared to other conventional topical dosage forms. The application of a film-forming spray to the skin is also more comfortable, with a more uniform distribution of the drug that forms a thin film following the texture of the skin or wound [17,18]. A film-forming spray must be prepared using polymers that are viscoelastic or have in situ film properties. One of the natural polymers that can form films in situ is chitosan, which has been proven to be an efficient drug carrier for wound-healing therapy due to its excellent mucoadhesive properties, and its antimicrobial and antioxidant activities [19].

Previous studies of hEGF-liposomes in acid-soluble chitosan hydrogels were investigated by Degim et al., who found that hEGF could be stable and last for a long period of time in the wound area [15,20]. However, the use of acids to dilute chitosan can produce distinctive aromas that are uncomfortable and can irritate wound mucosal tissue [21]. The application of hydrogel preparations also allows cross-infection, nonuniform dosage, and uneven drug distribution [22]. Therefore, we developed a film-forming spray using water-soluble chitosan containing hEGF-liposomes as a potential preparation for wound healing. In this study, we characterized the physicochemical properties, encapsulation efficiency, and sprayability of the film-forming spray of water-soluble chitosan (FFSWSC) containing hEGF-liposomes, and evaluated its effectiveness in healing wounds in mice.

## 2. Result

### 2.1. Excipient Optimization and the Preparation of Film-Forming Solutions

**Figure 1:**
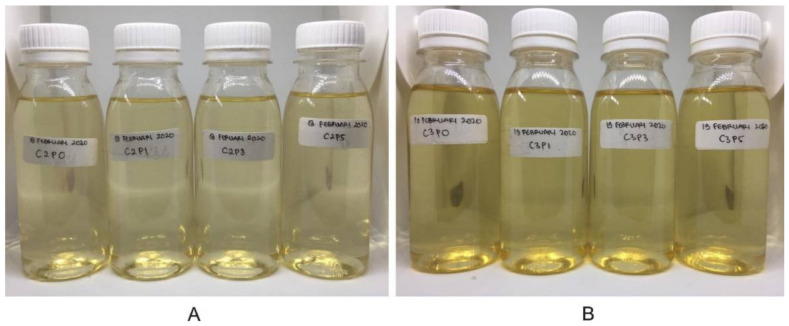
CWS 2% (**A**) and 3% (**B**) film-forming solutions using propylene glycol as the plasticizer.

Film-forming solutions produced using 1% CAS and 1% CWS had a clear yellow color, but the CAS has a sharp acetic acid aroma with a thicker consistency and was difficult to spray. Therefore, water-soluble chitosan was chosen as the basis for the film-forming solution. The CWS concentration was raised because it produced a rigid film and easily cracked at a level of 1%, while CWS was difficult to spray at a concentration of 4%. Therefore, the optimization was carried out at concentrations of 2% and 3%. Figure 1 shows the physical appearance of the optimized film-forming base solutions.

**Figure 2:**
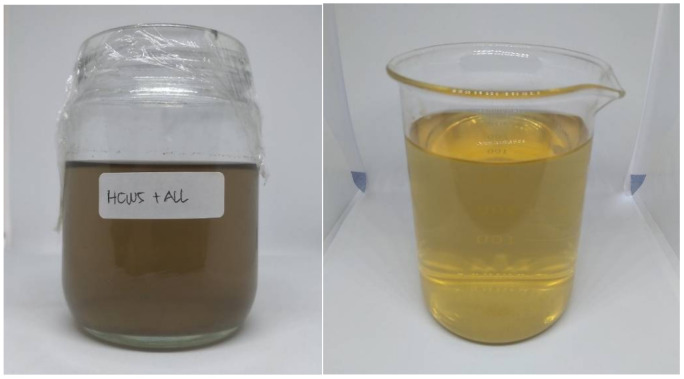
The film-forming solution containing methylparaben (**left**) and sodium benzoate (**right**).

The use of benzoic acid in the CWS solution resulted in white particles that were not dissolved at the bottom of the solution. The solubility of benzoic acid in aqueous solution is indeed very low [23]. With the use of methylparaben, blackening occurred after 40 days of storage at room temperature (Figure 2, left), while the base solution using the sodium benzoate preservative did not show significant physical changes. This phenomenon might have been caused by the interaction between methylparaben and tween 80. Therefore, the activity of methylparaben as a preservative decreased, and resulted in a growth of microorganisms [24,25]. The use of PG > 10% can prevent interactions between methylparaben and tween 80 [26,27]. However, the film-forming solution had poor sprayability and prolonged film-drying time, so the PG concentration used was less than 10%.

The use of glycerin as a plasticizer provided a significant increase in viscosity of the film-forming solution compared to PG. The drying time was also longer than for PG. Therefore, the optimization was continued using PG as a plasticizer and sodium benzoate as a preservative. The optimized formula can be seen in Table 1.

### 2.2. Preparation of hEGF-Liposomes

Lecithin and cholesterol were mixed into the chloroform:methanol (1:1) solvent to produce a mixture that dissolved and had the color of soy lecithin (like honey). As the mixture was stirred, the mixture developed a thicker consistency with a fading, paler color of lecithin. The mixture also had a bubbly texture. Therefore, the surface morphology of the lecithin–cholesterol film was observed using a digital microscope to confirm this phenomenon.

Then, the lecithin–cholesterol film that had dried was moistened with PBS pH 7.4, producing a yellowish-white suspension. The physical appearance of the liposome suspension obtained was similar to that obtained by Shashidar and Manohar [28], who also used soy lecithin as a source of phospholipids. The suspension was then immediately prepared for particle measurement and partly dried in a petri dish at room temperature for FTIR analysis.

### 2.3. Entrapment Efficiency

Based on the measurement results, a 99.87% entrapment efficiency of liposomes was found with a drug loading of 2%. This indicated that the liposomes had successfully coated the hEGF with a high entrapment efficiency. Further evidence was found through TEM observations and the therapeutic effectiveness.

### 2.4. Measurement of Viscosity and pH

The measurement results showed that the increase in chitosan concentration significantly affected its sprayability (spray angle and droplet size). It was found that a level of CWS of 3% with the highest concentration of propylene glycol (5%) resulted in a spray diameter that began to shrink (5.74 ± 0.26 cm) with a spray angle of 79.17 ± 0.48. This phenomenon showed that the optimal limit of viscosity to produce good sprayability was below 50 mPa.s. Ranade et al. [29] also confirmed that good sprayability was in the range of 25 to 45 mPa.s; this was shown at a CWS level of 4%, which produced a viscosity of 77.0 mPa.s with a centralized spray without spreading droplets, while the pH of chitosan ranged between 3.9 ± 0.1 and 4.4 ± 0.1.

### 2.5. Observation of hEGF-Liposome FTIR Spectrum

**Figure 3:**
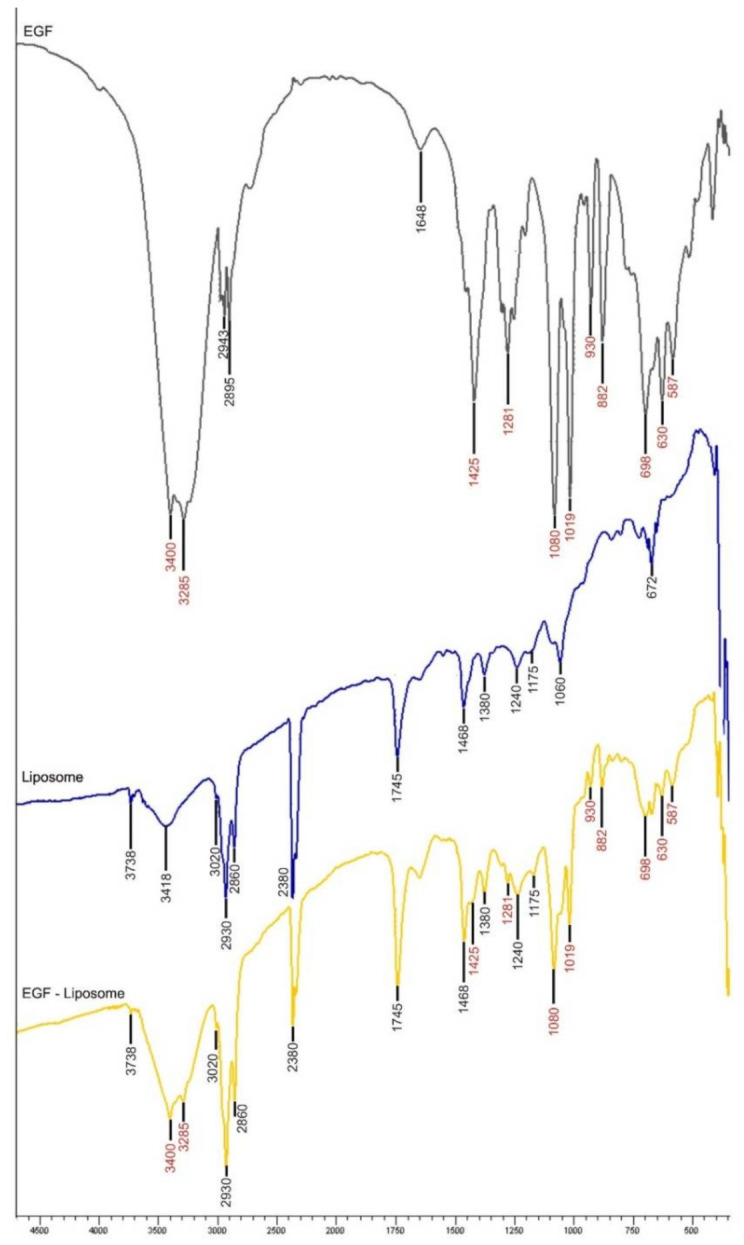
FTIR spectra of hEGF (**black**), liposomes (**blue**), and hEGF-liposomes (**yellow**) that did not show the presence of new covalent bonds in the encapsulation process.

Observation of the FTIR spectrum between hEGF and liposomes aimed to determine whether there was an interaction in the encapsulation of hEGF. The hEGF spectrum produced peaks at 3400, 3285, 2943, 2895, 1648, 1425, 1281, 1080, 1019, 930, 882, 698, 630, and 587 cm^−1^; whereas liposomes had peaks at 3738, 3418, 3020, 2930, 2860, 2380, 1745, 1468, 1380, 1240, 1175, 1060, and 672 cm^−1^. The FTIR spectrum of the hEGF-liposomes had a peak that is also found in the liposomes and hEGF itself (Figure 3). There were no unique peaks in the hEGF–liposome FTIR spectrum, which indicated that there was no formation of new covalent bonds in the encapsulation process. This phenomenon indicated that the release of hEGF from the liposomes was more efficient at the target site [28].

In the FTIR liposome spectrum, there was a peak at 3418 cm^−1^, indicating the presence of hydrogen bonds, which are the main character in macromolecular interactions such as those of phospholipids [30]. The peaks at 1240 and 1745 cm^−1^ were the corresponding peaks for the phosphate (P=O) and ester (C=O) groups on the polar head of phospholipids [31]. Aliphatic chains were also clearly seen at the peaks of 2930 and 2860 cm^−1^, followed by peaks at 1468 cm^−1^, which indicated the hydrophobic tail chain of phospholipids.

### 2.6. Determination of the Deacetylation Degree of Chitosan

**Figure 4:**
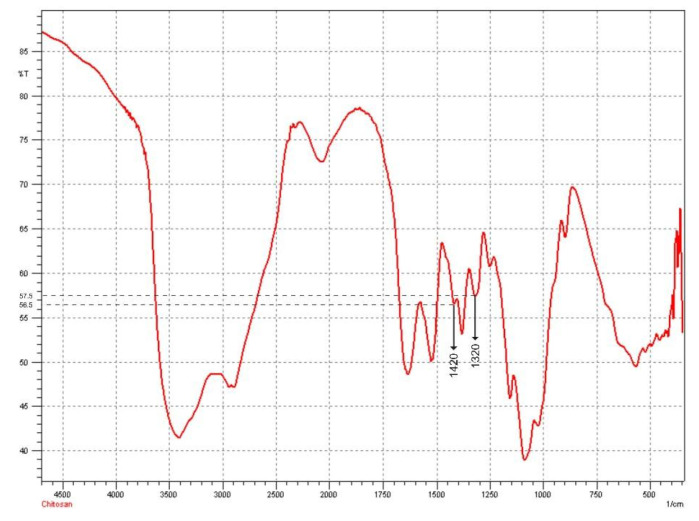
FTIR spectra of chitosan.

The %DD data from chitosan were used to confirm its relationship with the characteristics of the preparation. It was reported that high %DD could improve mechanical strength and the ability to absorb moisture from chitosan [32]. Based on the FTIR spectrum obtained (see Figure 4), the absorbance value was obtained by converting the transmittance value using the formula A = 2–log (% T) so that A_1320_ = 0.240; A_1420_ = 0.248; and %DD = 81.31%.

### 2.7. Determination of Particle Size, Polydispersity Index, and Zeta Potential

Different sonication processes produced different particle sizes. Sonication using a probe sonicator (cycle of 0.5 and amplitude of 70%) (Hielscher UP200S, Hielscher Ultrasonics GmbH, Teltow, Germany) produced a smaller particle size of the blank liposomes at the highest volume (16%), which was 64 nm, whereas the sonication bath process produced a larger size of 219.3 nm (see Table 2).

At the PDI value, the value that meets the requirements for lipid nanocarrier is below 0.3. Values above 0.7 indicate a very broad or inhomogeneous particle distribution [33]. In Table 2, it can be seen that the PDI values of the hEGF-liposomes indicated the uniformity of particle size distributions that met the requirements.

**Figure 5:**
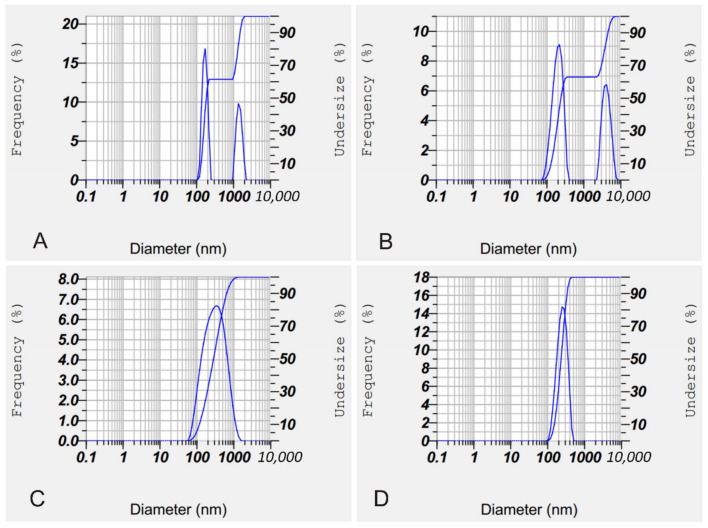
Graph of particle size of blank liposome KIT (**A**), hEGF–liposome KIT (**B**), blank liposomes test (**C**), and hEGF–liposome tests (**D**).

In Figure 5, it can be seen that KIT liposomes tended to be less stable, and formed aggregates. This was marked by the appearance of two peaks, one of which was nanosized, while the other was microsized. Microsized peaks were for a result of the aggregate size of the liposomes [34], whereas in the test liposome, only one peak was formed, and indicated that the dispersion was more stable than the KIT liposome. This was also supported by potential zeta data (see Table 2), where ethical zeta potential values ranged between ±30 and ±60 [35].

### 2.8. Observation of hEGF–Liposome Surface Morphology and TEM Analysis

**Figure 6:**
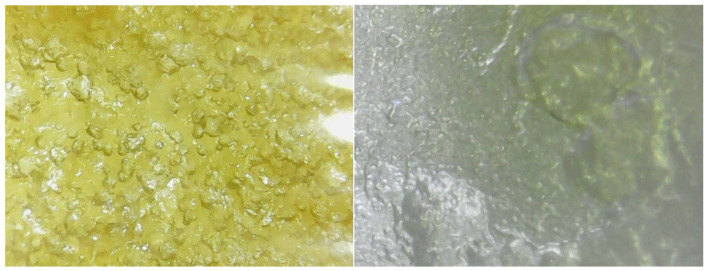
Observation of surface morphology using a digital USB microscope. Film of lecithin–cholesterol before being hydrated (**left**); hydrated hEGF–liposome film (**right**).

Morphological observations using a digital microscope at 1000× magnification confirmed the presence of bubbles that were absorbed in the mixture of lecithin and cholesterol during physical mixing (Figure 6). The stirring process was carried out slowly, but still produced a bubbly texture. This phenomenon showed the natural tendency of phospholipids to form vesicle structures in reaching thermodynamic equilibrium, which may be initiated by the humidity of the surrounding environment and the water content in the solvent (methanol) [36].

**Figure 7:**
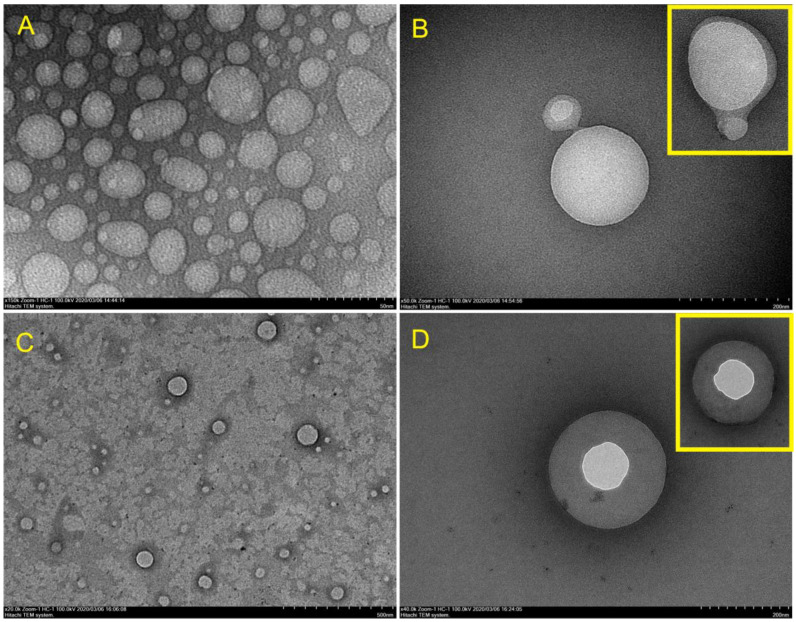
Shape observation using TEM: (**A**) empty KIT liposomes; (**B**) hEGF–liposome KIT; (**C**) empty test liposomes; (**D**) hEGF-liposome tests.

With the same preparation procedure, the test liposome and the KIT liposome were compared using TEM to observe the shape and the coating ability of hEGF. In Figure 7, it can be seen that the shape of the test liposome was more rounded (see Figure 7C), with a smaller size than the KIT liposome (see Figure 7A). Dispersions between KIT liposome particles were also close together, and its hEGF coating tended to form aggregates (see Figure 7B,D).

In contrast to the KIT liposome, the dispersed test liposome was more spaced, and formed a more rounded and stable coating. TEM observations also confirmed the results of particle size measurements using PSA that the particle size of the hEGF-liposome test ranged from 200–300 nm.

### 2.9. Occlusion Potential of Film

Occlusion factors describe a film’s permeability to water vapor. The smaller the occlusivity factor value, the better the permeability of the film to water vapor [29]. In this test, it was seen that an increase in CWS concentration correlated with an increase in the occlusivity factor (α = 0.47; *p* ≤ 0.05). Research conducted by Zhuang (2019) showed that the permeability of chitosan films to water vapor increased at %DD > 81.0% [37], and the chitosan used in this study had a %DD of 81.31%. Formulas that produce good water permeability contain 2% CWS.

### 2.10. Spray Angle, Pattern, and Droplet Size Distribution

Each spray produced a different spray pattern depending on the pattern of holes in the nozzle, but still produced a good spread. Spray angle showed the spread of spray from the film-forming solution. A good spray angle is below 85° [38,39]. The average spray angle formed ranged between 73.03 ± 1.96 and 79.17 ± 0.48 (see Table 3). Increasing the CWS concentration had a significant effect on increasing spray angle (α = 0.67; *p* < 0.01) and also on increasing droplet size (α = 0.39; *p* < 0.05). As previously explained, the viscosity of the solution greatly influenced the sprayability, and CWS had a greater role than propylene glycol in increasing viscosity. Statistical analysis also showed that the increase in propylene glycol concentration did not have a significant impact on increasing the spray angle and droplet size. Based on this test, the C2P3 formula showed the best results, which had the lowest spray angle and droplet size, with values of 73.03 ± 1.28° and 54.25 ± 13.34 µm, respectively.

### 2.11. Uniformity of Weight per Spray

Spray weights on all optimized formulas produced excellent uniformity, with an average weight of 0.14 ± 0.00 g to 0.15 ± 0.00 g, which indicated that the sprayer used could produce a uniform dose. Statistically, an increase in propylene glycol concentration showed a correlation with a decrease in spray weight (α = 0.33; *p* < 0.05).

When viewing all the test parameters (see Table 3), formulas containing 2% CWS and 3% PG (C2P3) produced the best film-forming spray characteristics, with the lowest spray angle and droplet size (73.03 ± 1.28° and 54.25 ± 13.338 µm) and the best spray weight uniformity.

### 2.12. Drying Shrinkage of the Film-Forming Solution

The test results showed that the film-forming solution of chitosan shrunk with a constant ratio for each volume to form a film; this was marked by the R^2^ value, which was equal to 1. These data can be used to predict the mass of the film that will be formed from the volume of the film-forming solution, which could be useful in planning industrial manufacturing and determining the dosage of drugs per unit mass of film.

### 2.13. Theoritical Film Thickness

The chitosan film (C2P3 formula) had a density of 1.04 g/cm^3^, an average film area of 65.86 ± 0.42 cm^2^, and an average spray weight of 0.14 ± 0.004 g. Based on these data, the film thickness formed by spraying was about 0.002 cm, or 20 microns.

### 2.14. Stability Test

The one-month stability test was carried out by storing the preparation in a climatic chamber in zone IVB conditions. During the test, there was no significant change in pH and viscosity (see Table 4). The particle size (about 200 nm) also did not change significantly. The PDI value changed from 0.22 to 0.26, but was still acceptable for a lipid nanocarrier (below 0.3) [33].

### 2.15. In Vivo Study

**Figure 8:**
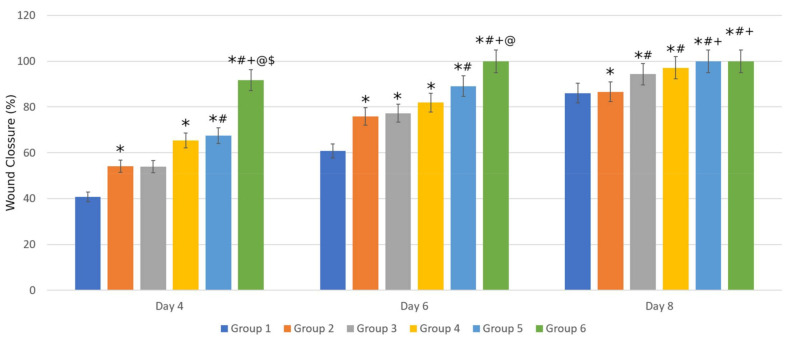
Wound closure percentage of each group and the significance of the differences analyzed using Games-Howell and Bonferroni post hoc tests. Note: (*, *p* < 0.05) shows a significant difference to group 1. (#, *p* < 0.05) shows a significant difference to group 2. (+, *p* < 0.05) shows a significant difference to group 3. (@, *p* < 0.05) shows a significant difference to group 4. ($, *p* < 0.05) shows a significant difference to group 5.

A wound-healing effectiveness test was aimed seat observing the effect of the active substance coating system and continuous release through film formation by chitosan on the rate of wound healing. The effectiveness between hEGF KIT and recombinant hEGF produced in the previous study was also compared in this study [40]. Based on the test results, it was found that group 6 had a significant rate of wound closure compared to all other groups, with wound closure reaching 91.671% on day 4. On day 4, groups 2, 4, and 5 showed a significant difference in wound closure compared to the control group. Group 5 also has a significant difference from group 2. This showed that hEGF and chitosan had natural wound-healing activity, and that coating hEGF using liposomes can increase the hEGF’s effectiveness. On day 6, the wounds in group 6 had closed completely, with a significant difference from all groups except in group 5. All groups showed a significant difference from the control group, with a percentage of wound closure in groups 2, 3, 4, 5, and 6 of 75.885%, 77.288%, 81.958%, 89.115%, and 100%, respectively. The other group achieved 100% wound closure on day 8 and above. This assay showed that hEGF could be more effective through coating with liposomes, and was significantly affected by the control of hEGF release by chitosan. The effectiveness of recombinant hEGF also appeared to be better than hEGF KIT, although not statistically significant (see Figure 8).

## 3. Discussion

The nature of the excipient used greatly determines the sprayability of a mixture. Ingredients that affect the viscosity and flow properties of the mixture need to be optimized to obtain the best formula. In this study, the effect of chitosan and PG on the sprayability of the mixture was studied. CWS at 2% and PG at 3% produced the most optimal spray angle, droplet size, spray weight uniformity, and occlusion factor. CWS concentration played an important role in determining the spray angle and droplet size, while PG affected the flow properties of CWS so that it reduced the spray weight uniformity. This was certainly not related to viscosity, because there was no significant difference in the spray weights of 2% and 3% CWS, while the viscosity was quite different. Chitosan is known to have thixotropic flow properties that can become more fluid when stressed, and can return to its original consistency after the stress is removed [41,42]. However, PG is reported to have a large influence on the nature of chitosan flow [43]. PG is also known to increase the stickiness and friability of chitosan films at 5% PG [39,44].

Sonication using a probe sonicator produced physical changes, with slowly blackened hEGF–liposome dispersion. This phenomenon may have been due to the high temperature generated by the probe sonicator, which can degrade hEGF [25,26]. In addition, using a probe sonicator for an extended period of time can cause the lipids to become de-esterified, and titanium on the probe can slough off and pollute the solution [27]. Therefore, the sonication process was carried out using a bath sonicator. A non-titanium probe sonicator with temperature controller may be suitable. In Table 2, it can be seen that the particle size of the hEGF-liposomes was smaller than that of the empty liposomes. In several studies, a decrease in liposome size was also shown after exposure to drugs or protein [45,46,47]. According to the Derjaguin–Landau–Verwey–Overbeek (DLVO) theory, this decrease in size can be caused by osmotic pressure, in which water is evacuated from the liposome nucleus due to changes in the difference in solute concentrations in the system and the liposome nucleus [47,48,49]. The size of the liposome KIT was also larger than the test liposome. This phenomenon could be influenced by the surface charge of the liposome, where ionic liposomes generally have a smaller size compared to neutral liposomes [46]. Based on the potential zeta value, it can be seen that the test liposome had a negative charge, while the KIT liposome had no charge (neutral). The zeta potential value obtained was also influenced by the use of tween 80 as a surfactant. This surfactant also played a role in maintaining the stability of the liposome dispersion.

The application of liposomes as an hEGF coating aimed to protect the hEGF from protease degradation. In this study, liposome-coated hEGF showed a better acceleration of wound healing than uncoated hEGF, although not statistically significant. In this assay, the group of mice treated with liposome-coated hEGF showed complete wound closure on day 8, while the group given uncoated hEGF showed complete wound closure on day 10 and above. Although liposome-coated hEGF had faster wound closure than without coating, continuous release control was still required to prevent receptor mediated endocytosis.

**Figure 9:**
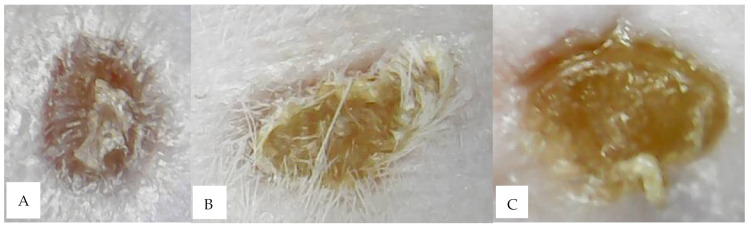
The different wound textures of the three groups: (**A**) hEGF–liposome; (**B**) hEGF–liposome–base; (**C**) base group.

In this study, chitosan played a role in regulating sprayability, and also formed a thin film matrix to regulate hEGF release. This role was important to keep hEGF levels continuously available to provide a sustainable therapeutic effect. Based on the in vivo test results, it could be seen that the administration of liposome-coated hEGF in a chitosan base significantly increased the rate of wound closure compared to other groups, with complete closure on day 6. In addition, the group that was given chitosan (groups 2 and 6) showed different characteristics of wound closure. Groups 2 and 6 tended to form scabs on the wound tissue, so that the wound became dry and closed quickly (see Figure 9). Scabs are said to be beneficial because they can prevent blood loss and infection. In addition, the scab also prevents exposure to ultraviolet radiation well [46]. Scabs in group 6 also did not appear to leave scars, and even the scar areas in some mice had regrown hair by day 9. Based on the test results, we concluded that liposome coating can increase the wound-healing activity of hEGF through protection against protease degradation and continuous release of chitosan, providing optimal levels of hEGF in the wound area constantly.

## 4. Material and Methods

### 4.1. Materials

We used acid-soluble chitosan (CAS) (food and medical grade with a deacetylation degree of >90%, made of crab shell or shrimp shell, Bio Chitosan, Indonesia), water-soluble chitosan (CWS) (food and medical grade with a deacetylation degree of >90%, made of crab shell or shrimp shell, Bio Chitosan, Indonesia), hEGF obtained from recombinant results from previous studies [40], soy lecithin (food and medical grade, Lansida, PT. Saraswanti Indo Genetech, Indonesia), cholesterol monohydrate (sigma grade with purity of ≥99%, made of sheep wool, Sigma Aldrich, St. Louis, MO, USA), liposome KIT (sigma grade, made of lyophilized egg yolk, L4395, Sigma Aldrich, USA), propylene glycol (PG) (PT. Brataco, Indonesia), glycerin (PT. Brataco, Indonesia), tween 80 (Merck, Kenilworth, NJ, USA), phosphate-buffered solution (PBS) (PT. Brataco, Indonesia), potassium bromide (Merck, Kenilworth, NJ, USA), methylparaben (Sigma Aldrich, St. Louis, MO, USA), acetic acid (Sigma Aldrich, St. Louis, MO, USA), benzoic acid (Sigma Aldrich, St. Louis, MO, USA), and sodium benzoate (Sigma Aldrich, St. Louis, MO, USA).

### 4.2. Excipient Optimization and Preparation of Film-Forming Solution (Base Solution)

The selection of polymers between CAS and CWS was based on their sprayability. Here, 5% PG and 5% glycerin were used as plasticizers; these were selected based on the characteristics of the resulting film. In addition, 2% tween 80 was used as a surfactant to increase the chitosan solubility and dispersion stability of the hEGF-liposomes, while 0.1% methylparaben, 0.1% benzoic acid, and 0.1% sodium benzoate were used as preservatives; these were selected based on solubility and compatibility between excipients.

The preparation of the base solution was achieved by dissolving CAS in a 0.5% acetic acid solution, and the CWS in distilled water was then homogenized using a stirrer for 1 h. Each chitosan solution (CAS and CWS) was mixed with tween 80. The plasticizer was added slowly. Then, the preservatives were finally added and stirred until homogeneous.

### 4.3. Preparation of hEGF-Liposomes

The liposome preparation was carried out using the thin-film hydration method. Cholesterol and phospholipids (soy lecithin) were mixed at a 1:1 molar ratio in 6 mL of chloroform:methanol (1:1). The solvent was then evaporated using a rotavapor at 50 °C. The hEGF solution in a phosphate buffer at pH 7.4 was then added to the dried liposome film with a loading dose of 2%. Then, the hEGF–liposome mixture was mixed until homogeneous at a speed of 3,000 rpm (50 °C) and then sonicated using a bath sonicator (Branson 5200 Ultrasonic, Branson, Brookfield, CT, USA) for 30 min [20].

### 4.4. Entrapment Efficiency

Standard curves were made using hEGF standard solution (1000 ppm) that was diluted to 25, 50, 100, 200, and 400 ppm. Each solution was put into the spectrophotometer’s microplate in an amount of 100 µL. The deposition of hEGF–liposome particles was carried out using a high-speed microcentrifugator (Benchmark’s MC-12, Benchmark Scientific, Inc., Sayreville, NJ, USA) at a speed of 13,500 rpm for 1 h. The supernatant was separated and then stored in a microplate in an amount of 100 µL. Bradford reagent was added to the microplate in an amount of 40 µL for each standard solution and sample solution. Absorbance measurements of standard and sample solutions were carried out at a wavelength of 595 nm (Epoch™ Microplate Spectrophotometer, BioTek Instrument, Inc., Winooski, VT, USA). The concentration of free hEGF in the sample solution was determined using the linear equation of the standard curve. The obtained linear equation can be seen below:

(1) y =5.341x +0.026

### 4.5. Preparation of Film-Forming Spray

The film-forming spray was prepared by mixing hEGF-liposomes into a base solution to a final hEGF concentration of 75 µg/mL using a mixer at a speed of 1000 rpm. After being homogenized, the mixture was sonicated for 30 min [20,50,51,52].

### 4.6. Measurement of Viscosity and pH

The viscosity of the base solution was measured using a Brookfield viscometer (NDJ-8S Viscometer, Dongguan Tianjian Machinery Equipment Co., Ltd., Changsha, China). The viscosity data were used to find the value of the limit of the viscosity of the film-forming solution that could be sprayed [53]. The pH of the solution was measured using a pH meter (HI 2211 pH/ORP Meter, HANNA Instruments, Woonsocket, RL, USA). Each measurement was made five times.

### 4.7. Observation of the hEGF–Liposome FTIR Spectrum

Interactions between hEGF and liposomes were analyzed using FTIR spectrophotometry (Shimadzu Prestige 21 FTIR spectrophotometer). Here, 200 mg KBr pellets were used as blanks, and 1 mg of hEGF–liposome film that had been dried at room temperature (25 °C) was analyzed by FTIR at wavelengths of 4000 cm^−1^ to 400 cm^−1^.

### 4.8. Determination of the Deacetylation Degree of Chitosan

The degree of chitosan deacetylation was determined using the FTIR chitosan spectrum by calculating the absorption ratio between a wavelength of 1320 cm^−1^, which is a typical peak of OH, NH_2_, or CO, and a wavelength of 1420 cm^−1^, as a typical peak of acetyl in chitin [54]. The following equation was used to calculate the deacetylation degree (DD) of chitosan:

(2) %DD=100−A1320A1420 −0.38220.03133

### 4.9. Determination of Particle Size, Polydispersity Index, and Zeta Potential

The determination of particle size, polydispersity index, and potential zeta of blank liposomes and hEGF-liposomes were carried out using a particle size analyzer (Horiba SZ-100, Horiba Ltd., Kyoto, Japan). The test was carried out by dispersing the sample in a phosphate buffer at pH 6.8, after which 1 mL was taken for testing [55].

### 4.10. Observation of hEGF–Liposome Surface Morphology and TEM Analysis

The lecithin–cholesterol film formed was observed using a digital microscope (M8704-1000, Chongqing Dontop Optics Co., Ltd, Chongqing, China) at 1000× magnification to observe the differences in surface morphology of the liposome film before and after being hydrated. Transmission electron microscopy (TEM) (HITACHI HT7700, Hitachi Ltd., Tokyo, Japan) was also used to observe the shape and successful coating of the liposome. We added 1% phosphotungstic acid (pH 6.0) to the sample, and then one drop was placed onto a carbon-coated copper grid. Samples were dried at room temperature, and then the surface morphology was observed.

### 4.11. Occlusion Potential of Film

This test was performed by covering the mouth of the glass beaker containing 50 mL of water with filter paper. One of the papers was sprayed with a film-forming solution and allowed to form a film. The glass beaker was then stored at room temperature and humidity. The permeability of the film to water was determined based on the reduced water weight in the beaker glass [29]. The assessment was determined using the following formula:

(3) $$F=\frac{A-B}{A}\times 100$$

where F is the occlusivity factor, A is the reduced water weight in the glass beaker with filter paper without film, and B is the reduced water weight in the beaker glass with a film-coated filter paper.

### 4.12. Spray Angle, Pattern, and Droplet Size Distribution

Water-sensitive paper was used to obtain a clear pattern down to the size distribution of the spray droplets. The nozzle used was an ordinal spray nozzle [22]. The diameter of the fixed pattern was then measured to determine the spray angle. Five repetitions of measurements were taken.

(4) Spray angle θ=tan−1lr

where l is the distance of the paper surface from the nozzle, and r is the radius of the circle. The spray spacing that is generally used is 15 cm [56]. The droplet size distribution was determined using ImageJ software (version 1.53e). The spray-pattern image was converted to a binary image, and then the average droplet size (µm) was measured.

### 4.13. Uniformity of Weight per Spray

The uniformity of weight per spray was determined by measuring the weight of each spray [29]. Spray weight was weighed at spray 5, 10, 20, 30, and 50 to determine the uniformity and repeatability of the dose [57]. In this test, the pressure applied on the sprayer was not controlled for mimicking the real usage.

### 4.14. Drying Shrinkage of the Film

This test was carried out to determine the reduction in weight during the drying process of the film. Several volume variations of the film-forming solution (1, 2.5, 5, 10, and 15 mL) were dried on a petri dish to obtain a linear equation:

(5) y =0.971x +0.0335

### 4.15. Theoritical Film Thickness

The film thickness was calculated using the following equation (ASTM E0252-06R13 standard):

(6) $$\mathrm{Thickness}\;\left(\mathrm{cm}\right)=\frac{\mathrm{Mass}\;\left(g\right)}{\mathrm{Area}\;\left({\mathrm{cm}}^{2}\right)\times \;\mathrm{Density}\;\left(g/{\mathrm{cm}}^{3}\right)}$$

### 4.16. Stability Test

The preparations were stored in a climatic chamber at a temperature of 40 ± 2 °C and RH 75 ± 5% according to the conditions of the IVB zone. Preparations were stored for 1 month, and changes in pH and viscosity were observed on days 0, 1, 7, 14, and 28. The particle size of the hEGF-liposomes was again tested to observe changes in particle size and distribution on the final day of observation.

### 4.17. In Vivo Study

#### 4.17.1. Animal Preparation

The inclusion criteria for the animals were male mice of the Swiss Webster strain aged 6–8 weeks with a body weight of 20–30 g, that were active and healthy during the adaptation period. The exclusion criteria were mice that were sick during adaptation, or had an infection or died during treatment [58]. Prior to testing, mice were acclimatized for 1 week before the experiment, with daily feeding and drinking and cleaning of cages twice a week on a regular basis. The population of test animals per group was calculated using the Frederer equation [59]. Based on this equation, the number of mice used was 24, with a number of mice per group of 4 mice. All experiments were performed following the guidelines of OIE animal welfare standards and were approved by the local Ethical Committee Faculty of Medicine, Universitas Padjadjaran, Bandung (No:1296/UN6.C10/PN2017).

#### 4.17.2. Animal Grouping and Wound Making

Mice were anesthetized with ketamine 40 mg/Kg BW intramuscularly, then placed on a surgical tub in the prone position. The hair around the middle back of the mice was shaved, then cleaned with alcohol-soaked cotton. The wound was made in a circular form with a diameter of 4 mm using a punch biopsy. The test animals were then grouped and marked as follows:

Group I: negative control (without any treatment);

Group II: given the water-soluble chitosan base solution;

Group III: given hEGF KIT solution (dose 75 µg);

Group IV: given recombinant hEGF solution (dose 75 µg);

Group V: given hEGF–liposome suspension (dose 75 µg);

Group VI: given FFSWSC containing hEGF-liposomes (dose 75 µg).

Groups II–VI were given the treatment at intervals of 2 days. Administration of the preparation at intervals of 2 days aimed to observe the effect of controlled release of the chitosan film matrix on the effectiveness of hEGF. The wound area was observed digitally using a camera (Canon EOS 1200D), and the wound area was calculated by ImageJ software until the wound was completely closed.

### 4.18. Statistical Analysis

The data were statistically analyzed using the Statistical Package for the Social Sciences (SPSS) version 22 (IBM Corporation, New York, NY, USA). The correlation between the variables of the dosage characteristics was analyzed using the Pearson correlation test, while the multiple comparison analysis in the in vivo test was carried out using a post hoc Games-Howell test for day 4 and a Bonferroni test for days 6 and 8.

## 5. Conclusions

The hydration film method produced hEGF-liposomes that were round and spread with a Z-average of 219.3 nm, whereas the film-forming solution, which provided good sprayability, was the formula containing 2% water-soluble chitosan and 3% propylene glycol, with viscosity, spray angle, droplet size, spray weight, and occlusion factor of 21.94 ± 0.05 mPa.s, 73.03 ± 1.28°, 54.25 ± 13.33 µm, 0.14 ± 0.00 g, and 14.57 ± 3.41%, respectively. The FFSWSC containing hEGF-liposomes was stable during storage for a month in an IVB zone condition. A wound healing activity test on mice showed that hEGF-liposomes in FFSWSC accelerated wound closure significantly, with a complete wound closure on day 6. Based on the findings, we concluded that FFSWSC containing hEGF-liposomes has the potential to be used as a wound dressing.

## Figures and Tables

**Table 1:** The optimized formulas.

Formula	Excipient (%)			
	Chitosan	Propylene Glycol	Tween 80	Sodium Benzoate
C2P0	2	0	2	0.1
C2P1	2	1	2	0.1
C2P3	2	3	2	0.1
C2P5	2	5	2	0.1
C3P0	3	0	2	0.1
C3P1	3	1	2	0.1
C3P3	3	3	2	0.1
C3P5	3	5	2	0.1

**Table 2:** The value of z-average, polydispersity index, and zeta potential.

Figure	Z-Average (nm)	Polydispersity Index	Zeta Potential (mV)
Empty liposome *	64	0.34	-
Empty liposome	338.9	0.41	−57.3
hEGF–liposome	219.3	0.22	−39.7
Empty liposome KIT	341.0	0.50	12.8
hEGF–liposome KIT	853.8	0.66	25.8

Note: all liposomes were prepared using a bath sonicator, except that marked with (*).

**Table 3:** Average values of measurements of viscosity, pH, spray angle, droplet size, and spray weight.

Formula	Viscosity (mPa.s)	pH	Spray Angle (θ)	Droplet Size (µm)	Spray Weight (g)	Occlusion Factor (%) *
C2P0	20.90 ± 0.06	4.3 ± 0.1	74.67 ± 1.96	92.90 ± 14.10	0.14 ± 0.00	12.44 ± 2.97
C2P1	21.00 ± 0.00	4.4 ± 0.1	77.05 ± 0.85	88.90 ± 46.04	0.15 ± 0.00	14.44 ± 5.26
C2P3	21.94 ± 0.05	4.2 ± 0.1	73.03 ± 1.28	54.25 ± 13.34	0.14 ± 0.00	14.57 ± 3.41
C2P5	23.46 ± 0.23	4.2 ± 0.1	75.51 ± 1.94	69.07 ± 12.05	0.14 ± 0.00	19.69 ± 3.79
C3P0	52.00 ± 0.00	3.9 ± 0.1	76.90 ± 0.84	82.55 ± 22.44	0.14 ± 0.00	25.26 ± 4.11
C3P1	53.18 ± 0.26	4.0 ± 0.1	77.87 ± 0.71	112.82 ± 44.70	0.13 ± 0.00	22.93 ± 4.42
C3P3	55.12 ± 0.24	4.0 ± 0.1	77.74 ± 0.83	117.15 ± 14.60	0.14 ± 0.00	22.28 ± 3.03
C3P5	56.20 ± 0.24	3.9 ± 0.1	79.17 ± 0.48	84.29 ± 37.44	0.14 ± 0.00	20.47 ± 1.46

Note: All tests were repeated five times, except those marked with (*), which had three repetitions.

**Table 4:** Stability data for the film-forming spray of water-soluble chitosan containing hEGF-liposomes.

Day	pH	Viscosity (mPa.s)	Particle Size (nm)	PDI
0	4.24 ± 0.05	21.94 ± 0.05	219.3	0.219
1	4.24 ± 0.05	21.74 ± 0.05	-	-
7	4.26 ± 0.05	21.62 ± 0.16	-	-
14	4.22 ± 0.05	21.60 ± 0.14	-	-
28	4.24 ± 0.05	21.58 ± 0.13	207.9	0.260

## Data Availability

Not applicable.

## Sample Availability

Lyophilized hEGF is available from the authors.

## Abbreviations

CAS	Acid-soluble chitosan
CWS	Water-soluble chitosan
DD	Deacetylation degree
DFU	Diabetic foot ulcer
GFs	Growth factors
hEGF	Human epidermal growth factor
PDI	Polydispersity index
PG	Propylene glycol
WVP	Water vapor permeability
FFS	Film-forming spray
FFSWSC	Film-forming spray of water-soluble chitosan
